# Genome-wide identification of GH17 family genes and their expression patterns associated with bud dormancy-regrowth regulation in *Prunus persica*

**DOI:** 10.3389/fpls.2025.1693135

**Published:** 2025-10-08

**Authors:** Xuehui Zhao, Haifeng Xie, Yifei Han, Jiaqi Zhang, Yihua Qi, Zejie Zhang, Maoxiang Sun, Meiying Liu, Fen Wang, Qingjie Wang

**Affiliations:** ^1^ School of Advanced Agricultural Sciences, Weifang University, Weifang, China; ^2^ College of Horticulture and Landscape Architecture, Yangzhou University, Yangzhou, China; ^3^ College of Agriculture and Forestry Science and Technology, Weifang Vocational College, Weifang, China; ^4^ Garden Greening Section, Binzhou City Bincheng District Public Utilities Service Center, Binzhou, China

**Keywords:** bud dormancy regulation, β-1,3 glucanase gene family, peach, expression pattern, PpKINβ2, PpMIEL1

## Abstract

**Introduction:**

β-1,3-glucanase (GH17), which is necessary for symplastic transport during dormancy establishment, maintenance, and release, plays important roles in regulating cell-cell communication through plasmodesmata. However, the comprehensive identification of GH17s in peach and their function in bud dormancy regulation is still poorly understood.

**Methods:**

Forty-eight *PpGH17* genes were identified from the peach genome and analyzed for phylogenetic relationships, conserved motifs, gene structure, domains, chromosomal location, syntenic relationships and cis-acting elements. Gene expression patterns were validated using qRT-PCR and protein interactions were confirmed by Y2H assays and BiFC assays.

**Results:**

These 48 PpGH17 members in peach were classified into three major clades, namely, α, β, and γ, and were distributed unevenly across eight chromosomes. *Cis*-element analysis of the *PpGH17* genes revealed that a large number of PpGH17 family members could respond to ABA or GA. By re-analyzing a RNA-seq data, ten *PpGH17* genes in peach and apricot showed significantly changed expression during the transition from endodormancy to ecodormancy. The expression patterns of the *PpGH17* genes during different dormancy stages in peach showed that these genes were closely associated with dormancy release and bud regrowth regulation. The GCC box-like element was found in the promoter sequence of eleven *PpGH17* genes, suggesting that they may be directly regulated by PpEBB1 and may participate in the bud-break stage. Y2H assays and BiFC assays showed PpGH17-8 can interact with PpKINβ2 and PpMIEL1.

**Discussion:**

Our study provides a foundation for understanding the functions of *PpGH17* genes in regulating peach bud dormancy. Furthermore, this study will facilitate the identification of key candidate genes for molecular breeding and genetic engineering efforts targeting dormancy-related traits in peach.

## 1 Introduction

Bud dormancy is an evolved strategy of perennial deciduous trees located in temperate regions to cope with harsh winter conditions, especially low temperatures. Before winter, the cessation of perennial deciduous tree growth is induced by shorter photoperiods or decreased temperatures in autumn, and the trees subsequently form a bud at the apex and enter endodormancy (Petterle et al., 2013). During the endodormancy stage, buds cannot sprout even under favorable conditions. Prolonged exposure to low temperatures is necessary for endodormancy release, and buds acquire the ability to sprout only after receiving adequate exposure to cold. However, the buds still display a dormancy phenotype at that time due to unfavorable conditions, such as low temperatures in winter, which is known as ecodormancy (Yang et al., 2021; Ding et al., 2024).

Studies have shown that plasmodesmata membrane-lined channels can change in response to environmental and developmental signals; are crucial for the active transmembrane transport of a large spectrum of molecules, including RNAs, proteins, hormones, and metabolites; and function in plant development, systemic signaling transport, and defense (Li et al., 2020; Schreier et al., 2023; Zanini et al., 2024). Studies have indicated that dormancy establishment and release are directly correlated with the closure and opening of plasmodesmata (Tylewicz et al., 2018). A transcriptome analysis revealed that many transcripts encoding plasmodesmata-associated proteins respond to short photoperiods (Tylewicz et al., 2018). Studies in birch, poplar, and Norway spruce have shown that short photoperiods stimulate callose deposition in plasmodesmata in the shoot apical meristem (SAM), leading to plasmodesmata closure and blocking intercellular communication, preceding dormancy establishment (Rinne et al., 2001; Lee et al., 2017; Tylewicz et al., 2018; Sun et al., 2019). Unlike poplar with thickened cell walls and plasmodesmata blocked by callose (Hu et al., 2024), open plasmodesmata were observed during winter in *Eucalyptus dunnii*, which are located in tropical/subtropical regions and undergo continuous cycles of growth in a year (Dai et al., 2023). After dormancy establishment, the accumulation of callose in plasmodesmata obstructs symplastic transport and limits the transport of growth-promoting signals into the SAM, such as FLOWERING LOCUS T (FT), a positive regrowth regulator that plays important roles in dormancy release (Rinne and van der Schoot, 1998; Ruonala et al., 2008; Rinne et al., 2011; Tylewicz et al., 2018). After prolonged exposure to low temperature, callose hydrolysis reopens closed plasmodesmata in dormant buds, leading to the import of FT and CENTRORADIALIS-LIKE 1 (CENL1) into the SAM and further accelerating bud dormancy release, which has been demonstrated in poplar and lily (Tylewicz et al., 2018; Pan et al., 2023). In addition, paradormancy is closely associated with cell-cell communication through plasmodesmata, as shown in a study on poplar, in which callose deposits were observed in paradormant axillary buds (AXBs) but strongly diminished before signs of bud regrowth (Rinne et al., 2016).

β-1,3-glucanases (glycosyl hydrolase GH17s, GH17s), which act on the decomposition of β-1,3-glucan polymer callose deposited in the neck regions of plasmodesmata (Lucas et al., 1993; Rinne et al., 2011; Wu et al., 2018), play important roles in regulating cell-cell communication through the plasmodesmata. GH17 families are relatively large, comprising approximately 50 and 100 members in *Arabidopsis* and *Populus trichocarpa*, respectively, and are phylogenetically grouped into three clades: the α, β and γ clades (Geisler-Lee et al., 2006; Doxey et al., 2007; Rinne et al., 2011). β-1,3-glucanases play roles in pathogen defense, cell division, pollen development, tube growth, the cold response, seed germination, and maturation, and 10% of them have a cell wall-related function that regulates plasmodesmata signaling, as reported in Arabidopsis and poplar (Geisler-Lee et al., 2006; Doxey et al., 2007). Disruption of the β-1,3-glucanase gene increased callose deposition and decreased the plasmodesmata permeability of embryo cells in Arabidopsis, leading to the inhibition of ABA synthesis genes, as intercellular signals, thus reducing seed dormancy (Wang et al., 2023). Several studies have demonstrated the GH17 proteins are closely related to bud dormancy regulation in deciduous trees. In poplar, the expression level of a *GH17* gene was downregulated under short photoperiod treatment (Tylewicz et al., 2018), whereas the production of β-1,3-glucanase was increased after sufficient chilling, which promoted callose degradation in the plasmodesmata channel and recovery of cell-cell transport, resulting in dormancy release, as reported in birch, poplar and tree peony (Rinne et al., 2001; Lee et al., 2017; Pan et al., 2023). A study in sweet cherry identified some candidate GH17s genes with quantitative trait loci mapping involved in bud dormancy and flowering time (Castède et al., 2015), and two *GH17* genes were identified as dormancy candidate genes due to their sharply changed expression during bud dormancy transition in *Prunus mume* (Zhang et al., 2018). The RNA-seq analysis in sweet cherry flower buds showed that differentially expressed *GH17* genes were significantly enriched during the various phases of dormancy (Fouché et al., 2023). A study analyzed transcriptome data from four *Prunus* species (almond, apricot, peach, and sweet cherry) at different dormancy stages, and found that endodormancy-related genes were significantly enriched with transcripts associated with cell wall modifications, indicating that cell wall remodeling is highly correlated with bud dormancy regulation in *Prunus* (Calle et al., 2022). However, few studies have focused on the regulation of bud dormancy by *GH17* genes in peach, and the research on *GH17* in peach has mainly centered on aspects such as fruit ripening and disease resistance (Pei et al., 2019; Rodríguez-Pires et al., 2020; Tsalgatidou et al., 2024).

During bud endo- to ecodormancy transition in *Prunus* species, differentially expressed genes associated hormones were significantly enriched, especially abscisic acid (ABA) and gibberellin (GA) (Canton et al., 2021; Calle et al., 2022), which antagonize plasmodesmata callose turnover to regulate callose degradation in plasmodesmata and control intercellular trafficking (Veerabagu et al., 2023). Studies have indicated that *GH17* genes also participate in phytohormone-regulated dormancy mechanisms. Exogenous GA can promote dormancy release. A study in birch revealed that GA_3_ and GA_4_ strongly differ in their ability to induce a canonical bud burst, which may be partly due to their selective effects on the expression of specific sets of *GH17* genes (Rinne et al., 2011). Bioactive GA_4_ upregulates α‐clade 1,3‐β‐glucanases that localize to plasmodesmata, where they decompose callose, whereas applied ABA downregulates several GA_3_/_4_‐inducible α‐ and γ‐clade 1,3‐β‐glucanases that hydrolyze callose at plasmodesmata, thereby increasing plasmodesmata callose accumulation (Veerabagu et al., 2023). In hybrid aspen, ABA can suppress glucanases that break down callose, leading to the blockage of plasmodesmata (Tylewicz et al., 2018).

In this study, we identified the GH17 family members in peach by the genome-wide analysis and characterized the phylogenetic relationships, chromosome distributions, syntenic relationships, conserved motifs, gene structures, and promoter *cis*-acting elements of the *PpGH17* genes. Additionally, to investigate whether *PpGH17* genes are involved in bud dormancy regulation, the relative expression patterns of the *PpGH17* genes were analyzed during the different dormancy stages, suggesting that many genes were associated with dormancy release and bud regrowth. Finally, Y2H assays and BiFC assays confirmed that PpGH17–8 interacts with PpKINβ2 and PpMIEL1. Yet, the *GH17* genes in bud dormancy regulation of peach is still poorly understood, our results screened *PpGH17* genes crucial for bud dormancy regulation, which will help to elucidate the biological functions and identify potential key genes regulating bud dormancy-regrowth in peach.

## 2 Materials and methods

### 2.1 Plant materials

The peach (*Prunus persica*) cultivar Zhongyou 4, which was planted in Shandong, Tai’an, China (36°18’N, 117°13’E), was used in this study. Leaf bud samples grown on first-year branches were collected approximately every half month from October until March of the following year. The samples were then immediately flash frozen in liquid nitrogen and subsequently stored at −80°C for subsequent experiments.

### 2.2 Identification of *GH17* genes from peach and phylogenetic analysis

Glycoside hydrolase 17 (GH17) family members were identified using the online hmmsearch tool (https://www.ebi.ac.uk/Tools/hmmer/search/hmmsearch) by scanning the Hidden Markov Model (HMM) profile of the GH17 domain (Accession: PF00332) from Pfam (http://pfam-legacy.xfam.org/) in the *Prunus persica* online database using the default e-value. The obtained candidate GH17 members were further determined by the NCBI Conserved Domains Database (NCBI CDD) online tool (https://www.ncbi.nlm.nih.gov/Structure/bwrpsb/bwrpsb.cgi), and the members without the GH17 domain and the repeating sequences were removed.

All sequences of peach (*Prunus persica*) (Verde et al., 2013), poplar (*Populus trichocarpa*) (Tuskan et al., 2006) and *Arabidopsis thaliana* (Cheng et al., 2017) were downloaded from Phytozome (https://phytozome-next.jgi.doe.gov/). The isoelectric points (pIs) and molecular weights (MWs) of the PpGH17s were predicted by the online tool ExPASy (https://web.expasy.org/compute_pi/). The online subcellular location tool WOLF PSORT (https://www.genscript.com/wolf-psort.html) was used to predict the subcellular locations of 48 PpGH17 family members.

The full-length amino acid sequences of Arabidopsis, poplar, and peach were downloaded from Phytozome and used to construct a neighbor joining (NJ)-based unrooted phylogenetic tree after multiple alignment with ClustalW using MEGA ver. 6.0 software. The phylogenetic tree was based on the poisson model, with pairwise deletion of gaps, and evaluated via the bootstrap method with 1000 replicates.

### 2.3 Conserved motifs, gene structure and domains analysis

Conserved motif analysis of the PpGH17 proteins was performed by the online MEME tool (https://meme-suite.org/meme/tools/meme). The gene structure annotation information file of peach was downloaded from Phytozome for gene exon–intron structure analysis. The conserved domain was predicted by the NCBI CDD. The Gene Structure View (Advanced) tool of the TBtools software was used to visualize the information of genes, including the phylogenetic trees, conserved motifs, domains and gene structure (Chen et al., 2020).

### 2.4 Analysis of the chromosomal location, syntenic relationships and *cis*-acting elements in *PpGH17* genes

The Gene Location Visualize option of the GTF/GFF tool of the TBtools software was used to map the *PpGH17* genes onto specific chromosomes. The Dual Synteny Plot option of the MCScanX tool and the Multiple Synteny Plot tool of the TBtools software were used for syntenic analysis of the *PpGH17* genes in peach and Arabidopsis. The 2000 bp upstream sequences of the initiation codons (ATG) of the *PpGH17* genes were extracted and submitted to the PlantCARE online tool (https://bioinformatics.psb.ugent.be/webtools/plantcare/html/) to identify the *cis*-acting elements.

### 2.5 Total RNA isolation, cDNA synthesis and quantitative real-time PCR

Total RNA was isolated by the RNAprep Pure Plant Kit (polysaccharide- and polyphenolic-rich) (Tiangen, Beijing, China) according to the supplier’s instructions. The RNA quality was examined using a NanoPhotometer P360 (Implen, Munich, Germany) and 1.0% agarose gel electrophoresis. Reverse transcription was performed using a HiScript III 1st Strand cDNA Synthesis Kit (+gDNA wiper) (Vazyme, Nanjing, China). qRT–PCR was performed using ChamQ Universal SYBR qPCR Master Mix (Vazyme, Nanjing, China) in a 20 μL reaction volume following the thermal cycling conditions described in the supplier’s instructions. Threefold repetition was carried out for each cDNA sample. The 2^-ΔΔCt^ method was used to calculate the qRT–PCR data, which were normalized against the expression of the reference gene *Ppactin*. The relative expression levels of genes obtained from qRT–PCR were first averaged across three biological replicates, and then preprocessed via min-max normalization ((*x* - *x_min_*)/(*x_max_* - *x_min_*)), which linearly transformed the averaged expression values to a range of 0 to 1. The normalized expression matrix was visualized as a heatmap using Heml ver. 1.0 software. The sequences of primers used for qRT–PCR are listed in **Supplementary Table 1**.

### 2.6 Acquisition of RNA-seq data

Two sets of RNA-seq data were used in this study, both of which were obtained from public databases. The genes with fold change>2 and *p*-value<0.05 were identified as differentially expressed genes (DEGs). One of them was derived from the paper published by Yu et al. (2020). In that paper, the RNA-seq data showed that during the transition from endodormancy to ecodormancy, 1367 and 2102 DEGs were identified in apricot and peach trees, respectively. We used this set of RNA-seq data to identify differentially expressed *GH17* genes during the transition from endodormancy to ecodormancy. The other set of RNA-seq data was derived from the paper published by Zhao et al. (2020). In that paper, *EBB1* from peach transformed into poplar, and the RNA-seq data revealed the DEGs between *PpEBB1*-overexpressing (*PpEBB1*-*oe*) poplar and wild type poplar. We used this set of RNA-seq data to identify differentially expressed *GH17*genes in *PpEBB1-oe* poplar.

### 2.7 Yeast two-hybrid assays

The Y2H assays were performed using the Matchmaker Gold Yeast Two-Hybrid System (Clontech, Dalian, China). The CDS of *PpGH17-8* (forward primer, 5’-ATGGCATCGCTTTCACA-3’; reverse primer, 5’-TGTATTTCCACCAGGGTAAGT-3’) was cloned into a pGBKT7 vector, and the CDSs of *PpMIEL1* (forward primer, 5’-ATGGTTAGACAGAACAAAATGGAG-3’; reverse primer, 5’-TCAGCCTCTCGTTTGGC-3’) and *PpKINβ2* (forward primer, 5’-ATGGGGAATGTGAATGGG-3’; reverse primer, 5’-TTATCTCTGCAAGGACTTGTAGAGA-3’) were cloned into the pGADT7 vector, respectively. Recombinant vectors were transformed into Y2H Gold yeast. The yeast strains were cultured on -Trp/-Leu, -Leu/-Trp/-His/-Ade, and -Leu/-Trp/-His/-Ade media with X-α-gal at 30°C for 3 d. Each Y2H assay was performed in three independent biological replicates.

### 2.8 Bimolecular fluorescence complementation assays

The CDSs of *PpGH17–8* were cloned into the pSPYNE (Y^N^) vector, while the CDSs of *PpMIEL1* and *PpKINβ2* were cloned into the pSPYCE (Y^C^) vector, respectively. All recombinant vectors were transformed into *Agrobacterium tumefaciens* strain GV3101. For BiFC assays, agrobacterial suspensions (PpGH17-8-Y^N^ with PpKINβ2-Y^C^, PpGH17-8-Y^N^ with PpMIEL1-Y^C^) were mixed at a 1:1 volume ratio, and the mixture of PpGH17-8-Y^N^ with the empty Y^C^ vector as a negative control. These mixtures were used to infect onion epidermal cells for 30 min respectively, which were then incubated on MS medium at 28°C in darkness for 48 h. YFP fluorescence was observed using a laser-scanning confocal microscope (LSM880) (Zeiss, Oberkochen, Germany) with 514 nm. Each BiFC assay was performed in three independent biological replicates.

## 3 Results

### 3.1 Identification of PpGH17 family members and phylogenetic analysis in peach

After removing the members without the GH17 domain and the repeating sequences, 48 *GH17* genes were identified in peach in our study. The lengths of these GH17 proteins ranged from 203 aa (Prupe.7G245500) to 978 aa (Prupe.7G227000). Their predicted pI values ranged from 4.58 (Prupe.7G200500) to 9.61 (Prupe.7G051900). Their molecular weights (MWs) ranged from 22667.15 Da (Prupe.7G245500) to 109617.49 Da (Prupe.7G227000) (**Supplementary Table 2**).

**Figure 1:**
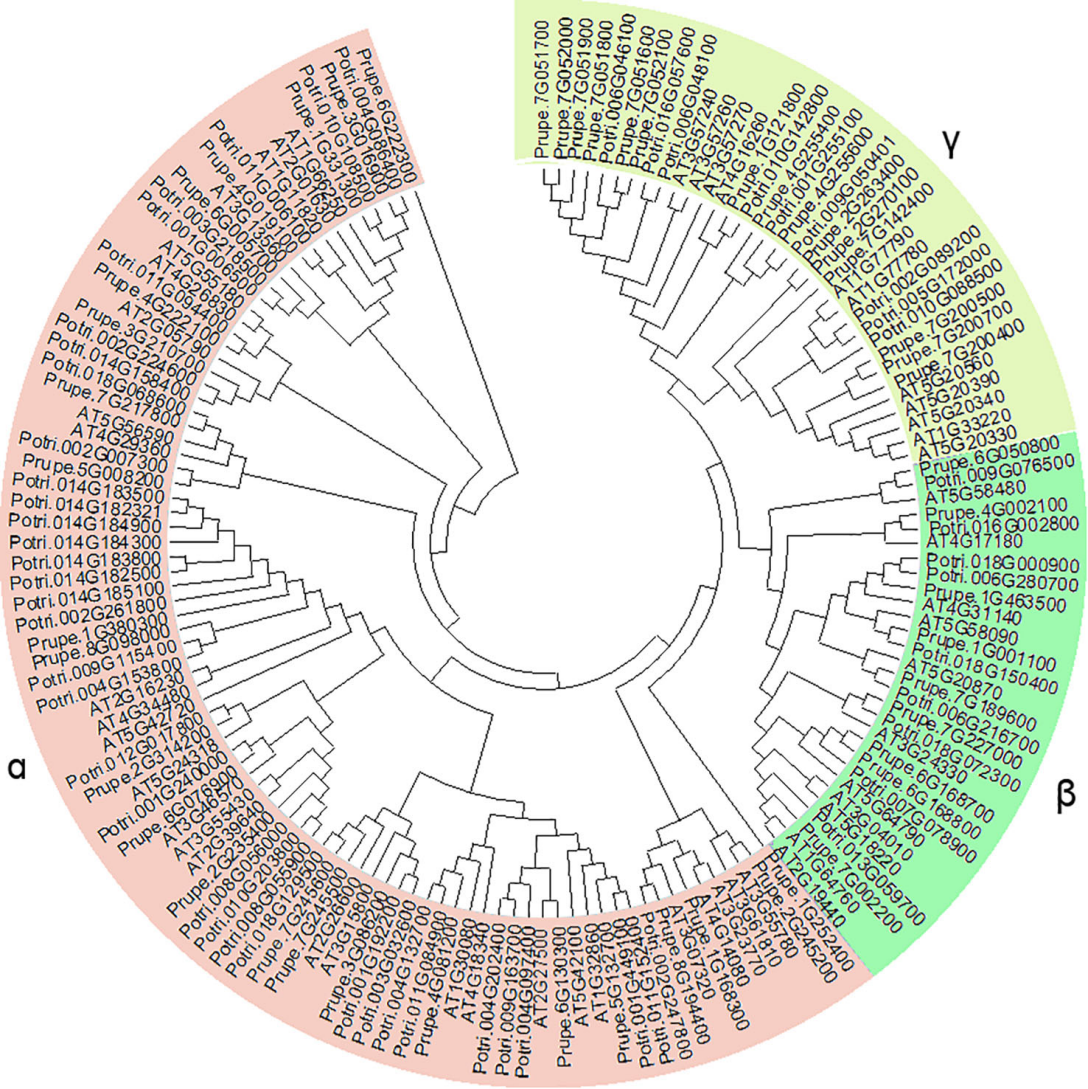
Phylogenetic analysis of GH17 family genes in peach, Arabidopsis, and poplar.

An unrooted phylogenetic tree was generated using the 48 *PpGH17* genes, together with 50 members in Arabidopsis and 54 in poplar, the models of herbaceous and woody plants, respectively (**Figure 1**), using MEGA6.0. In total, 152 proteins were subdivided into 3 major clades, α, β, and γ. Among the 48 PpGH17 family members, 24 belonged to clade α, 9 belonged to clade β, and 15 belonged to γ. Most PpGH17 members have a closer evolutionary relationship with poplar than with Arabidopsis.

### 3.2 Chromosome distribution and syntenic analysis of PpGH17 members

**Figure 2:**
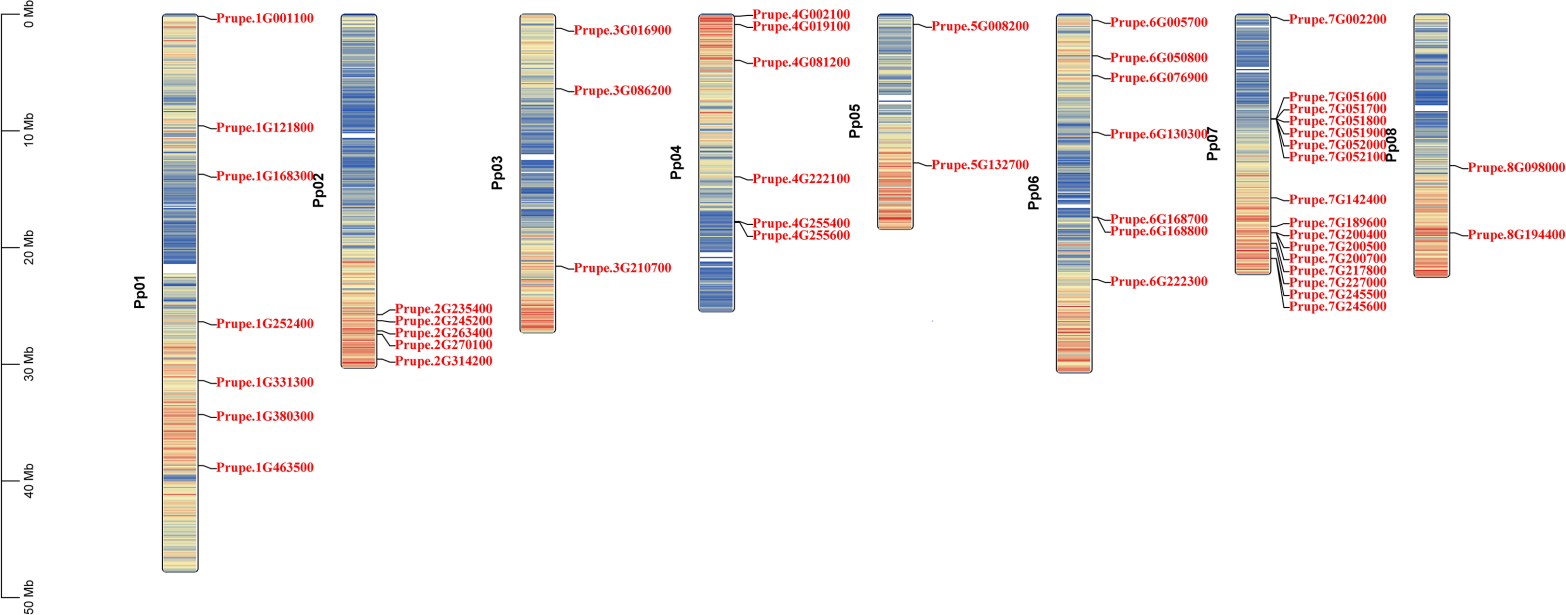
Chromosomal localization of 48 PpGH17 family genes.

The 48 PpGH17 members were distributed unevenly on chromosomes 1-8 (**Figure 2**). Chromosome 7 contained the most PpGH17s, with sixteen members. Chromosome 1 and chromosome 6 harbored seven members, followed by chromosome 4, with six members. Three PpGH17 members were located on Chromosome 3. Chromosomes 5 and 8 had the fewest, with two members each. Five *PpGH17* genes were located at the bottom of Chromosome 2.

**Figure 3:**
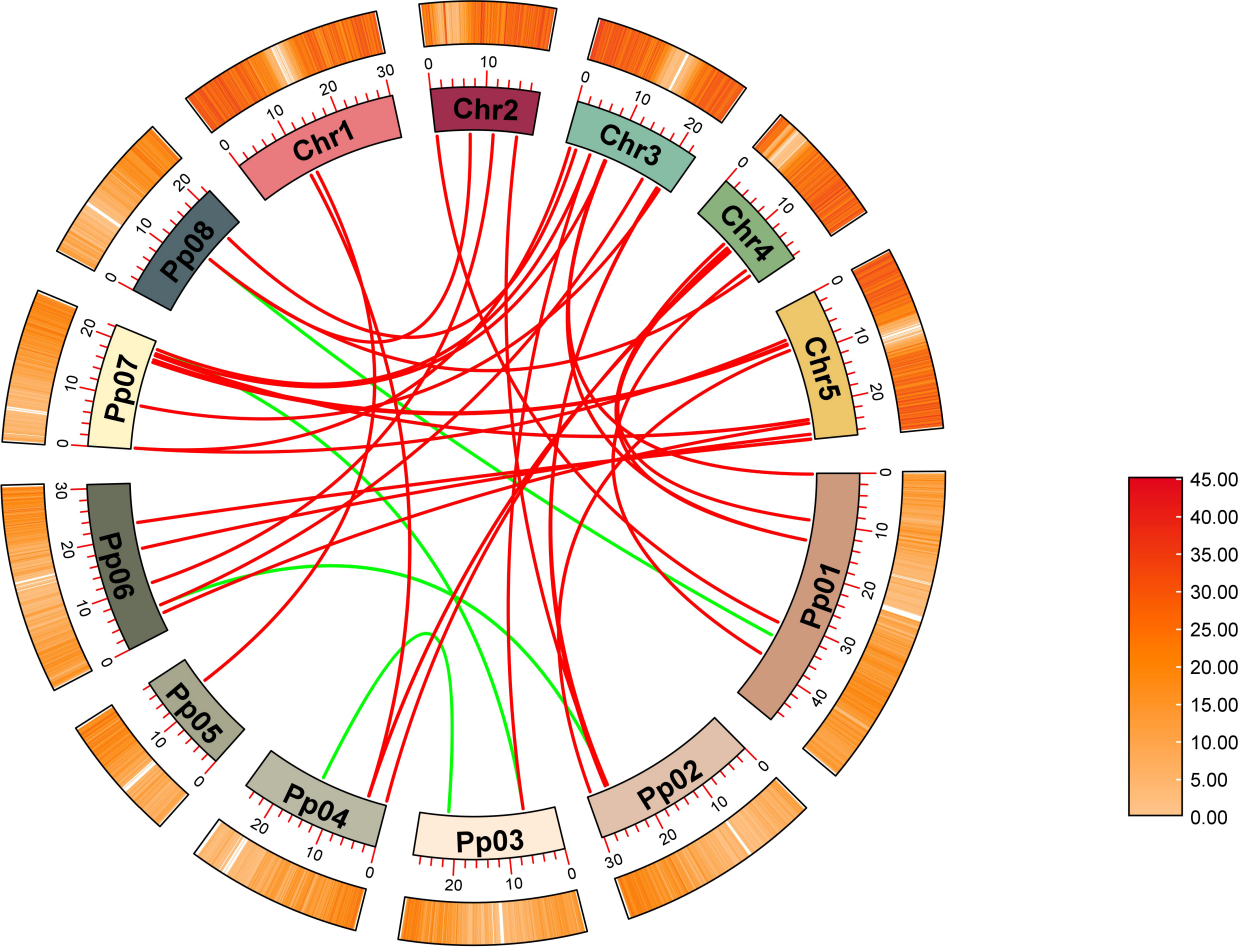
Synteny analysis of GH17 family genes in peach and Arabidopsis. Eight chromosomes from peach (Pp01–Pp08) and five from Arabidopsis (chr1–chr5) were identified.

The syntenic relationships between peach and Arabidopsis were analyzed to identify homologs. In total, 31 pairs of orthologous *GH17* genes were detected (**Figure 3**; **Supplementary Table 3**), including 29 *AtGH17*s and 26 *PpGH17*s. Most *PpGH17* genes matched one orthologous *AtGH17*, and only five *PpGH17*s (*Prupe.1G168300*, *Prupe.2G235400*, *Prupe.4G081200*, *Prupe.7G002200*, *Prupe.8G098000*) matched two orthologous *AtGH17*s. In addition, four paralogous pairs were observed among the 48 identified *GH17* genes in peach, revealing proteins corresponding to Prupe.3G086200 and Prupe.7G245500, Prupe.1G380300 and Prupe.8G098000, Prupe.3G210700 and Prupe.4G222100, or Prupe.2G235400 and Prupe.6G076900.

### 3.3 Domains, conserved motifs and gene structure analysis of PpGH17 members

**Figure 4:**
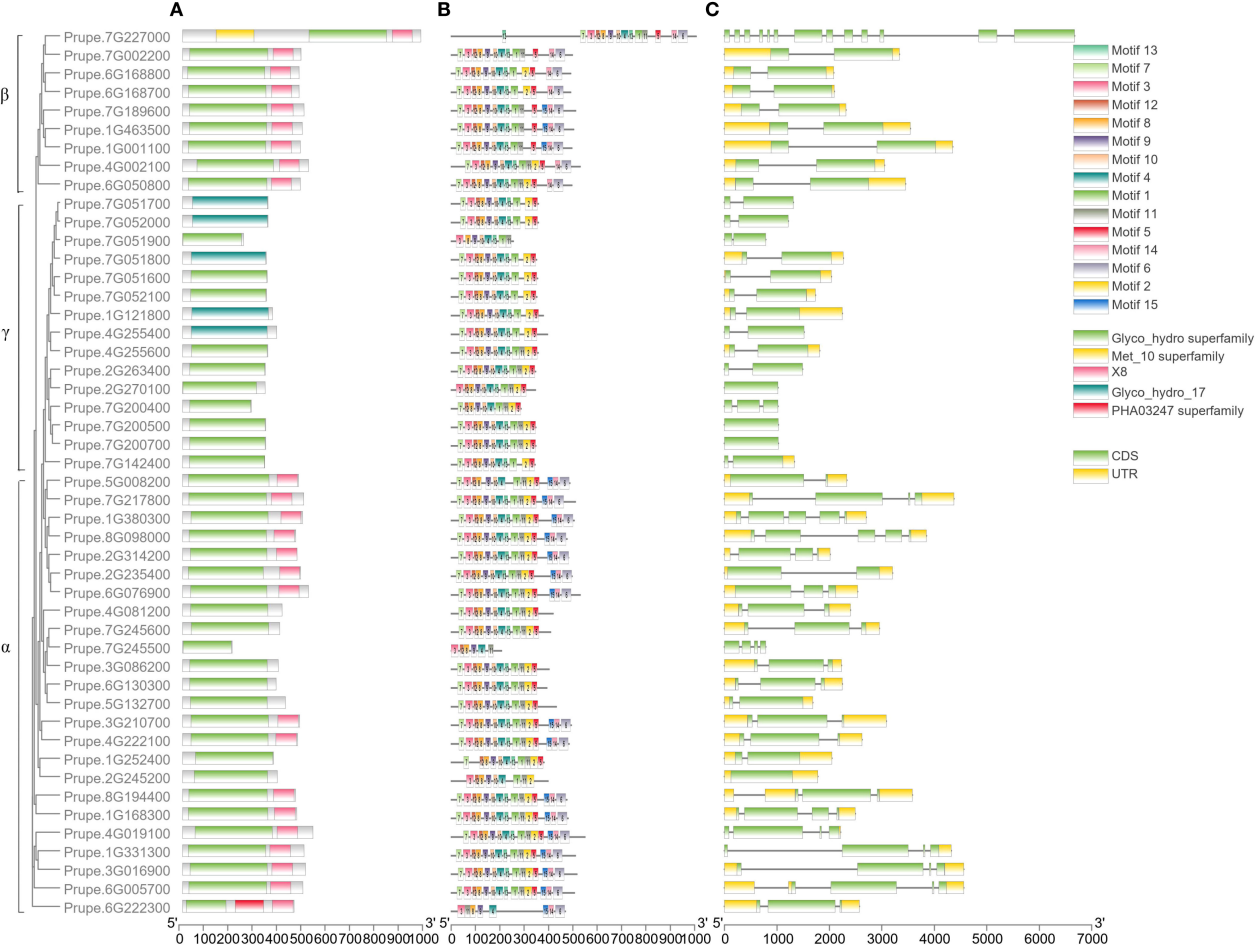
Domains **(A)**, conserved motifs **(B)** and gene structural analyses **(C)** of PpGH17 members.

All the GH17s contained a Glyco hydro superfamily domain or a Glyco hydro 17 domain (**Figure 4A**). All 9 β-clade members and 17 α-clade members contained an X8 domain at the C-terminus of the sequence. In addition, a Met 10 superfamily domain and a PHA03247 superfamily domain were found in Prupe.7G227000 and Prupe.6G222300, respectively, indicating that they may have special functions compared with other members.

The conserved motifs of 48 PpGH17s were analyzed, and 15 distinct conserved motifs were found (**Figure 4B**; **Supplementary Table 4**). Motifs 4, 8, and 9 were present in all 48 PpGH17 members, and motif 8 contained highly conserved glycine catalytic residues, which were present in all the members. Motifs 6, 14 and 15 are located in the C-terminus of the GH17 protein, present in 9 β-clade members and 17 α-clade members, while absent in the γ clade members. The motifs in different PpGH17 family members were highly consistent; generally, they could be divided into 2 main types, including or not including motifs 6, 14, and 15. These results indicate that the functions of the PpGH17 members may be highly similar.

The gene structure of 48 PpGH17s was analyzed using their coding DNA sequence (CDS) and genomic sequence (**Figure 4C**). A total of 34 genes presented both 5’ and 3’ UTRs, 3 members presented only 3’ UTR, and the remaining 11 genes presented no UTRs. In addition, the numbers of introns and exons in different genes were quite different. A total of 23 members presented 1 intron, 9 members presented 2 introns, 8 members presented 3 introns, and 3 members presented 3 introns. PpGH17 members in clades β and γ contained fewer introns than did those in clade α; all of the clade β members contained 1 intron, except for Prupe.7G227000, which contained 12 introns; most of the clade γ members contained 1 intron; and most of the clade α members contained 2 or more introns.

### 3.4 Promoter *cis*-acting element analysis

**Figure 5:**
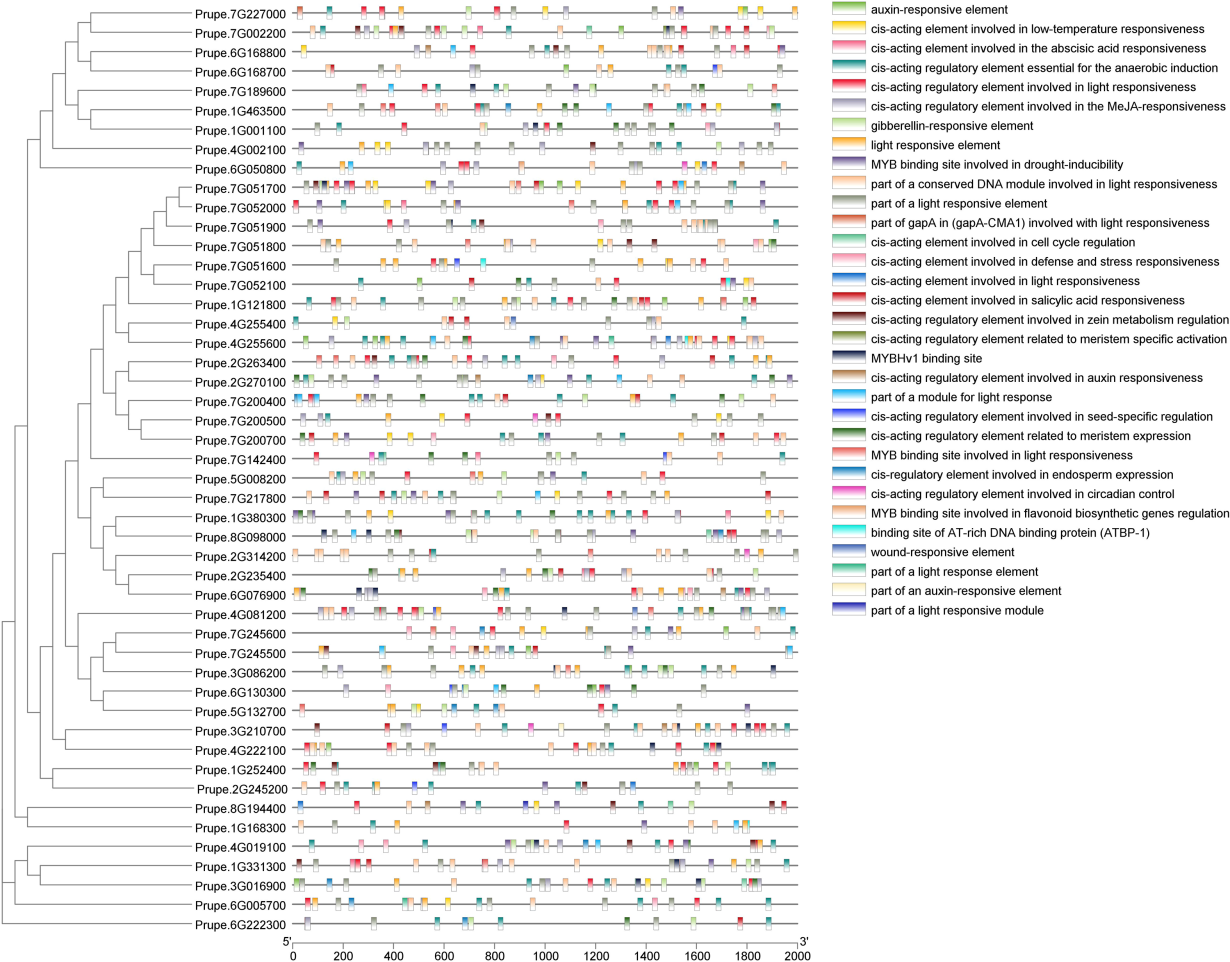
Promoter *cis*-element analysis of *PpGH17* genes.

To further infer the function and mechanism of the PpGH17 family, the upstream 2000 bp promoter sequences of ATG were used for *cis*-acting element analysis through the online PlantCARE tool. In addition to the unnamed and basic CAAT and TATA boxes, a total of 32 *cis*-acting elements were identified, with a focus on those relevant to dormancy regulation-particularly hormone responses (**Figure 5**; **Supplementary Table 5**). Notably, ABA response elements and GA response elements, the central hormones to bud dormancy regulation, were widely distributed in the PpGH17 family. ABA-responsive elements were present in 39 members, while GA-responsive elements were detected in 26 members. Other hormone-responsive elements, including Aux, SA, and MeJA were also identified in 22, 23, and 33 PpGH17 members, respectively. Each PpGH17 family member can respond to one to five types of hormones. In addition to hormone responses, other elements were detected, including those related to environmental stimuli, such as light and low temperature, MYB binding sites, and plant growth and development, such as meristem expression, and cell cycle. Light response elements were existed in all PpGH17 members, and low-temperature response elements were present in 21 members, which potentially reflecting coordination between environmental cues and dormancy transitions.

### 3.5 Expression levels of *GH17* family genes in floral buds of peach and apricot varieties at ecodormancy compared with those at endodormancy

**Figure 6:**
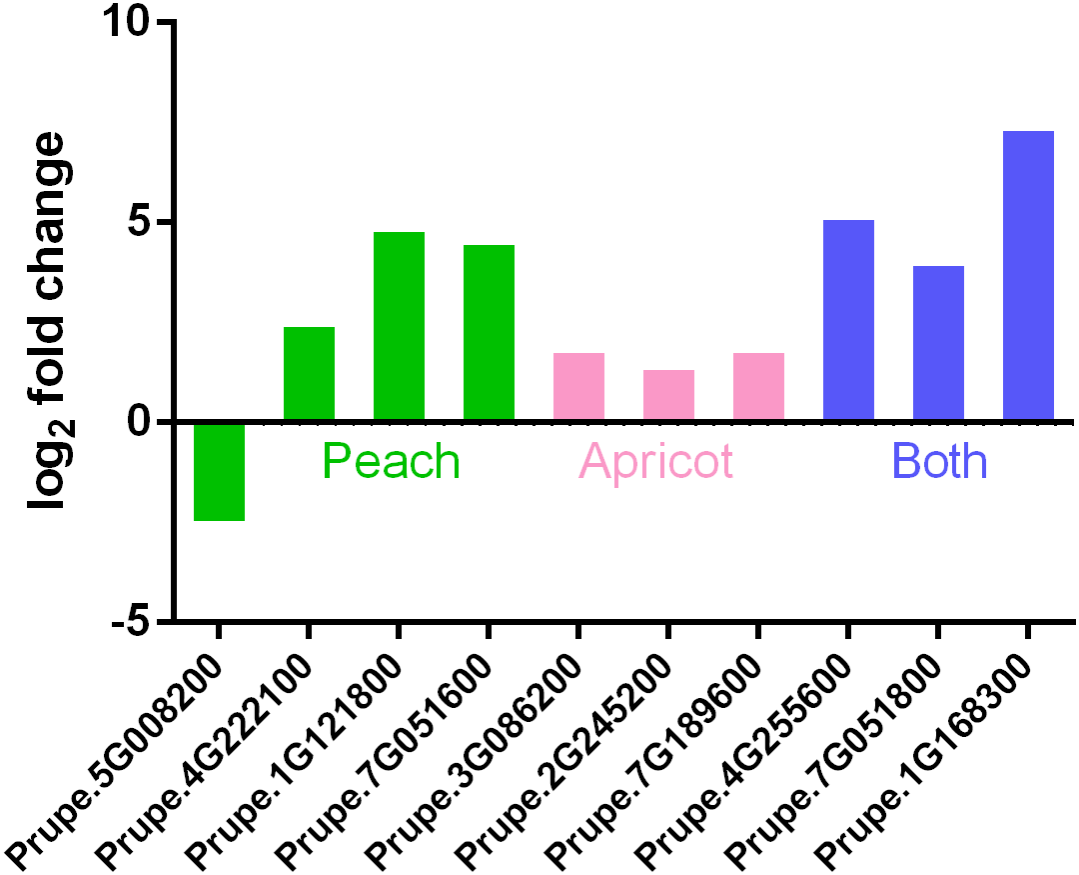
Differentially expressed *PpGH17* genes of apricot and peach trees during the transition from endodormancy to ecodormancy.

Among 1367 and 2102 differentially expressed genes during the transition from endodormancy to ecodormancy in apricot and peach, respectively, ten belonged to the *GH17* family, four of which were in peach, including *Prupe.5G008200 (PpGH17-13)*, *Prupe.4G222100 (PpGH17-14)*, *Prupe.1G121800 (PpGH17-40)*, and *Prupe.7G051600 (PpGH17-43)*; three were in apricot, including *Prupe.3G086200 (PpGH17-3)*, *Prupe.2G245200 (PpGH17-23)*, and *Prupe.7G189600 (PpGH17-29)*; and three were in both peach and apricot, including *Prupe.1G168300 (PpGH17-16)*, *Prupe.4G255600 (PpGH17-42)*, and *Prupe.7G051800 (PpGH17-45)* (**Figure 6**). The transcriptome profile results revealed that the expression of all the *GH17* genes was upregulated except for *Prupe.5G008200 (PpGH17-13)* at the ecodormancy stage compared with that at the endodormancy stage, indicating that most of the *GH17* genes have a positive function in regulating dormancy release.

### 3.6 Relative expression levels of the *PpGH17* genes during the bud dormancy stage

**Figure 7:**
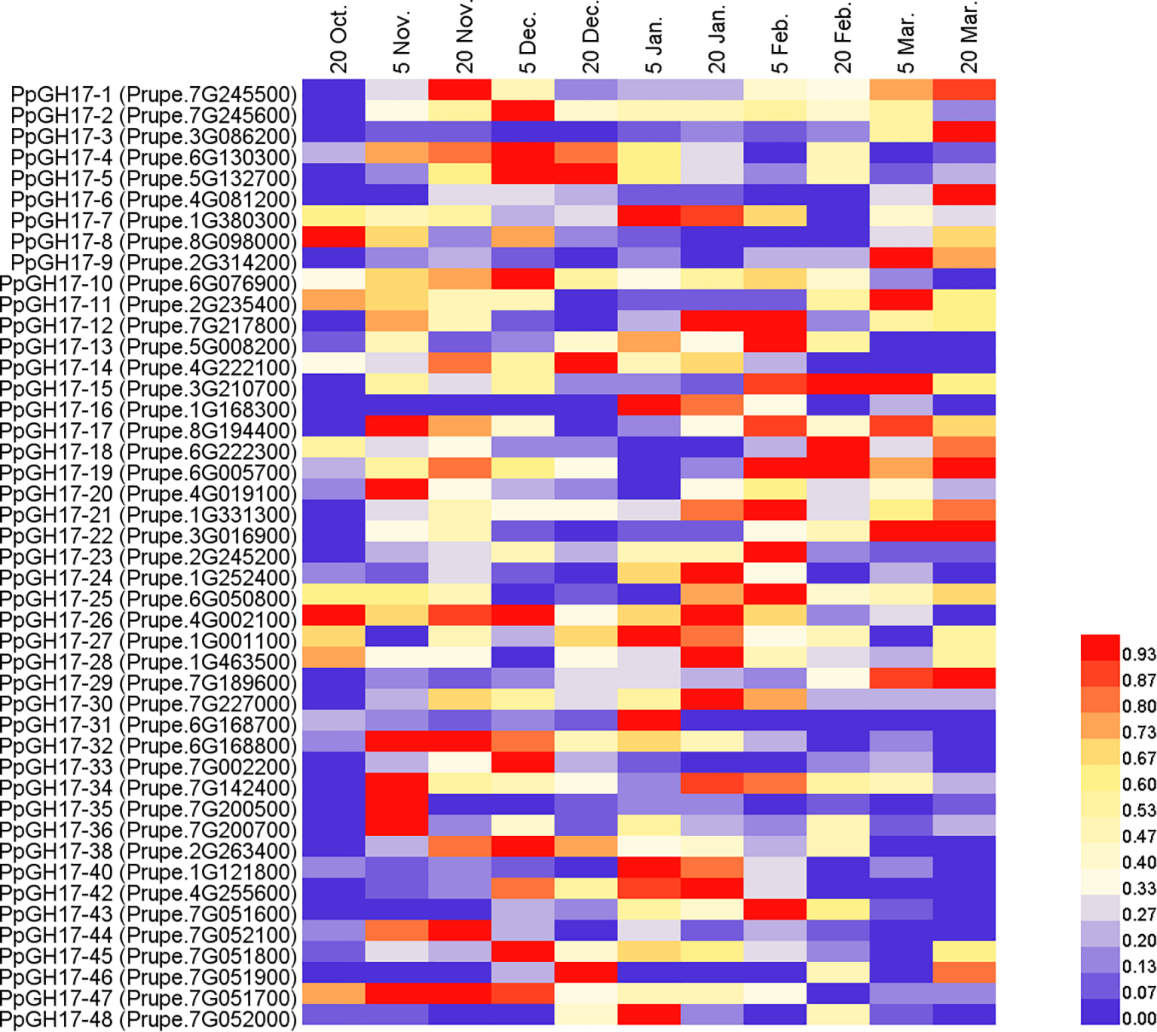
Heatmap of the differential expression of *PpGH17* genes in peach buds from the dormancy stage to the bud-break stage.

As described in our previous study, the buds entered the dormancy release stage on December 20th, clearly swelled on March 5th of the following year, and then burst on March 20th. To determine whether PpGH17 members participate in the dormancy process of peach buds, the relative expression of all 48 *PpGH17* genes was detected from endodormancy to bud break, and altered expression levels were observed (**Figure 7**; **Supplementary Figure 1**). During the dormancy release stage, the expression of some *PpGH17* genes, such as *PpGH17-2*, *PpGH17-4*, *PpGH17-5*, *PpGH17-6*, *PpGH17-10*, *PpGH17-19*, *PpGH17-22*, *PpGH17-30*, *PpGH17-33*, *PpGH17-34*, and *PpGH17-38*, first increased but then decreased. During the bud-break stage, the expression levels of *PpGH17-1*, *PpGH17-3*, *PpGH17-6*, *PpGH17-8*, *PpGH17–22* and *PpGH17–29* increased, whereas those of *PpGH17-10*, *PpGH17-13*, *PpGH17-34*, *PpGH17–43* and *PpGH17–48* decreased. These results indicated that the *PpGH17* genes may participate in different bud development stages.

### 3.7 *PpGH17* genes may be regulated by PpEBB1

In *PpEBB1*-*oe* poplar, 16 *PtGH17* genes were identified (**Supplementary Table 6**). Among them, 13 *PtGH17* genes were upregulated in *PpEBB1-oe* poplar, and three were downregulated. The differential expression of the *PtGH17* genes in *PpEBB1-oe* poplar suggested that *GH17* genes may be regulated by PpEBB1. Therefore, the sequences 2000 bp upstream of the ATG promoter of 48 *PpGH17* genes were analyzed. Eleven of them contained a PpEBB1 binding element, GCC box-like element (**Supplementary Table 7**). These results suggest that *PpGH17* genes may participate in the bud-break stage and are directly regulated by PpEBB1.

### 3.8 PpGH17–8 interacts with PpKINβ2 and PpMIEL1

**Figure 8:**
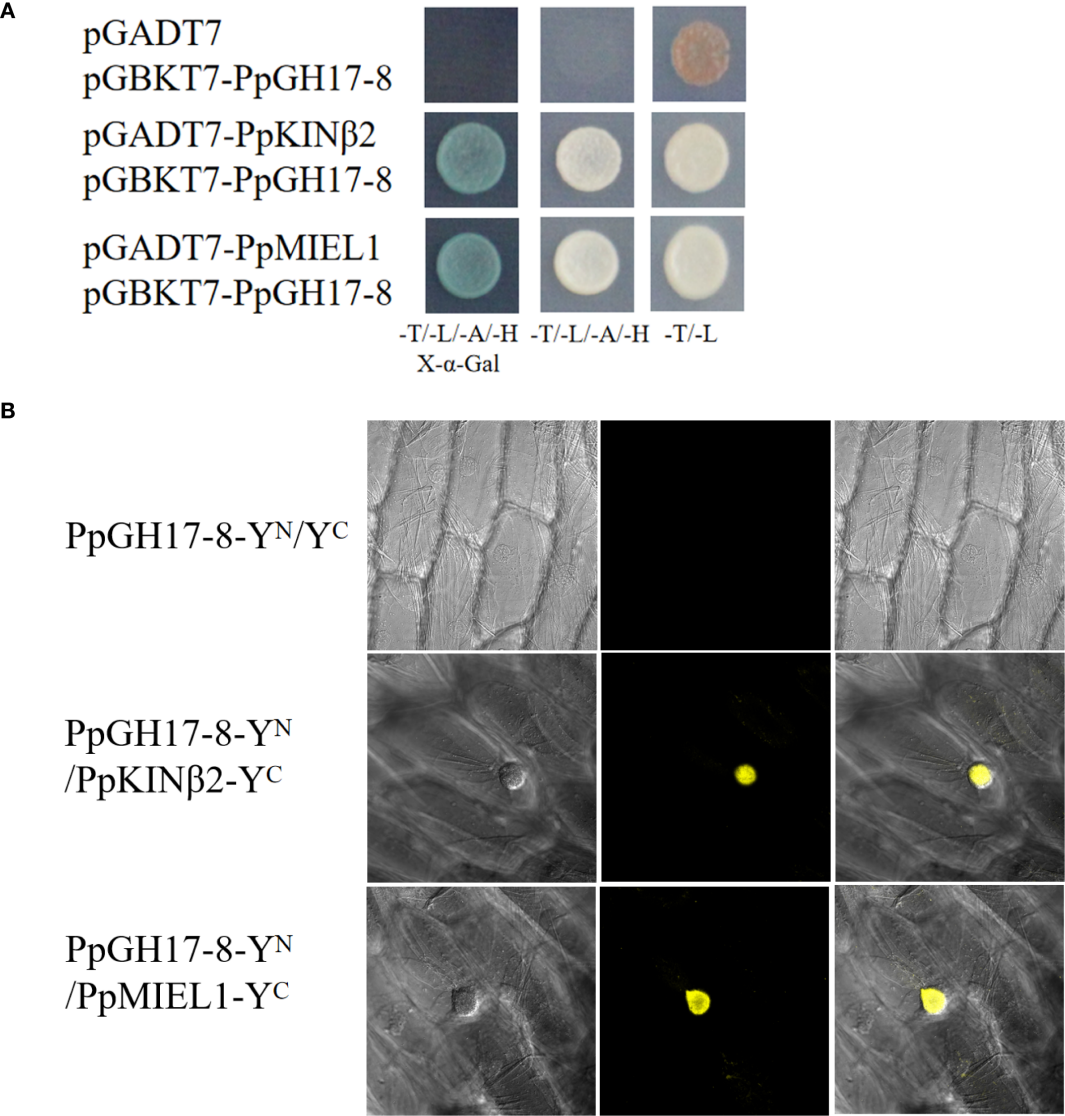
PpGH17–8 interacts with PpKINβ2 and PpMIEL1. **(A)** Interaction between PpGH17-8, PpKINβ2 and PpMIEL1 in the Y2H assays. **(B)** Interaction between PpGH17-8, PpKINβ2 and PpMIEL1 in the BiFC assays.

PpGH17–8 was selected for further exploration based on its expression trend during the dormancy process of peach buds. We used Y2H assays to screen for interacting proteins of PpGH17-8, identifying a *PpKINβ2* (*Prupe.4G004500*) gene and a *PpMIEL1* (*Prupe.1G465000*) gene, encoding a sucrose non-fermenting 1 (SNF1) related protein kinase 1 (SnRK1) regulatory β subunit and a RING domain E3 ligase, respectively. Results showed that yeast strains co-transformed with pGADT7-*PpKINβ2/*pGBKT7-*PpGH17-8*, or pGADT7-*PpMIEL1/*pGBKT7-*PpGH17–8* exhibited normal growth in the selective media (-Leu/-Trp/-His/-Ade, and -Leu/-Trp/-His/-Ade supplemented with X-α-gal) and turned blue in the medium supplemented with X-α-gal, while the negative control containing empty pGADT7 vector and recombinant pGBKT7-*PpGH17–8* vector could not grow on the selective medium (**Figure 8A**), suggesting PpGH17–8 can interact with both PpKINβ2 and PpMIEL1.

BiFC assays were performed to confirm the interactions between PpGH17–8 and PpKINβ2 or PpMIEL1 *in vivo*. Strong YFP fluorescence signals were detected in onion epidermal cells co-infiltrated with the recombinant vector combinations PpGH17-8-Y^N^/PpKINβ2-Y^C^ and PpGH17-8-Y^N^/PpMIEL1-pY^C^ (**Figure 8B**), while no YFP fluorescence was observed in the negative control, which infiltrated with PpGH17-8-Y^N^ and the empty Y^C^ vector. BiFC assays further proved that PpGH17–8 can interact with both PpKINβ2 and PpMIEL1.

## 4 Discussion

β-1,3-glucanase gene family 17 is a large and highly complex family that has been identified in many species and is involved in a series of biological functions in plant growth and development processes (Doxey et al., 2007; Pervaiz et al., 2021; Wang et al., 2024). Among the three clades of PpGH17 family, the α clade had the greatest number of members, whereas the β clade had the fewest members, which was consistent with studies in grape (Wang et al., 2024; Shi et al., 2025). The X8 domain, consisting of motifs 6, 14 and 15, exists in over half of the α-clade members and all the β-clade members, which is also defined as CBM43 and is responsible for binding β-1,3-glucan at plasmodesmata (Barral et al., 2005; Simpson et al., 2009); whereas all the γ-clade members lack the X8 domain, indicating that they may have functional distinctiveness. A study on paradormancy in hybrid aspen confirmed their distinct expression patterns during apical growth, AXB development, AXB paradormancy, and decapitation-induced AXB activation, that α-clade 1,3-β-glucanases were significantly upregulated by decapitation, whereas γ-clade 1,3-β-glucanases were downregulated, and the overexpression of an α-clade member induced an acropetal branching pattern, whereas all lines overexpressing a γ-clade member presented apical deviations (Rinne et al., 2016).

### 4.1 *Cis*-acting elements of *PpGH17* suggest that the involvement in ABA-GA interplay to regulate bud dormancy

The types of *cis*-elements of peach *GH17* genes in our study are highly similar to those of grape genes, for example, light, low temperature, GA and ABA-responsive elements were also identified in grape, suggesting that the functions and regulatory mechanisms of *GH17* genes are relatively conserved (Wang et al., 2024). *Cis*-acting elements in *PpGH17* promoters provide insights into their regulatory roles in bud dormancy, particularly through interactions with ABA and GA, the key antagonistic regulators of dormancy induction, maintenance, and release (Liu and Sherif, 2019; Veerabagu et al., 2023; Ding et al., 2024). The prevalence of ABA-responsive elements and GA-responsive elements strongly suggests that *PpGH17* genes are tightly integrated into ABA-GA signaling networks regulating dormancy. During bud dormancy establishment, SD-mediated decreases in GA levels or increases in ABA levels can trigger the closure of plasmodesmata (Singh et al., 2016). Studies have indicated that blockage of symplastic intercellular communication through plasmodesmata is essential for dormancy and occurs downstream of ABA-mediated control of dormancy in response to shorter photoperiods and ABA-insensitive hybrid aspen plants not only fail to establish bud dormancy but also cannot induce the closure of plasmodesmata (Tylewicz et al., 2018). Compared with those in WT plants, the upregulated expression of plasmodesmata-related genes such as *GH17–39* was altered in ABA-insensitive hybrid aspen plants under a short photoperiod, and the frequency of plasmodesmata closure was decreased in ABA-insensitive hybrid aspen plants (Tylewicz et al., 2018). The expression of γ‐clade glucanases in hybrid aspen is positively correlated with increasing ABA levels in developing buds (Veerabagu et al., 2023). These results indicate possible links between ABA, β-1,3-glucanases, and plasmodesmata closure and dormancy establishment (Gao et al., 2021). The application of GAs appears to eliminate low-temperature accumulation to accelerate dormancy release (Yuxi et al., 2021). GH17 members can be differentially induced by GA_3_ and GA_4_; GA_3_‐induced GH17 enzymes belonging to the γ-clade are upregulated during prolonged chilling, whereas GA_4_‐induced GH17 enzymes belonging to the α-clade are induced by growth‐supporting temperatures (Rinne et al., 2001, 2011; Hu et al., 2024). GA levels are closely associated with callose deposition, and GAs can potentially recruit *GH17* genes to reopen symplastic paths, release dormancy, and drive bud burst in hybrid aspen (Rinne et al., 2011). Together, these observations indicate that *PpGH17* genes likely act at downstream of ABA-GA crosstalk, mediating callose metabolism and plasmodesmata communication to regulate dormancy transitions.

Beyond ABA and GA, other phytohormones also have been found modulate dormancy by regulating plasmodesmata-mediated trafficking through the deposition or removal of callose in the dormancy process (Ding et al., 2024). Exogenous auxin application before bud break results in the removal of callose from sieve tubes and leads to the restoration of symplastic paths in magnolia (Aloni and Peterson, 1997). While breaking dormancy, MeJA treatment upregulates the expression of four genes encoding β-glucosidase (Jacobsen et al., 2013), and JA probably has opposite effects to ABA in the regulation of dormancy release (Ding et al., 2024). SA treatment can induce the secretion of a β-1,3-glucanase into the apoplast in transgenic Arabidopsis plants (Zavaliev et al., 2013). Collectively, these findings confirm that various phytohormones converge on callose metabolism and plasmodesmata function as key nodes for regulating the dormancy process.

### 4.2 *GH17* genes participate in bud dormancy regulation through callose hydrolysis in plasmodesmata

The transition from dormancy to growth revival of perennial woody plants is closely associated with the permeability of the plasmodesmata channel in the cell walls of buds and is caused by callose deposition and removal at the plasmodesmata, which is determined by callose synthase and 1,3-β-glucanase (Levy et al., 2007; Rinne et al., 2011; Ding et al., 2024). Therefore, *GH17* genes may play important roles during the bud dormancy process by regulating callose deposition in the plasmodesmata of the cell wall directly. During the bud dormancy induction stage, the expression of *GH17–39* in poplar, which participates in callose removal (Tylewicz et al., 2018), is downregulated, indicating that *GH17* genes may negatively regulate dormancy establishment. However, a previous study also revealed that in the early SD-induced stage, around the same time as the narrowing of the SAM plasmodesmata, *GH17* transcript levels increase, which is considered to lead to increased callose production caused by the upregulation of callose synthase transcripts (Ruonala et al., 2008; Rinne et al., 2011).

Previous studies have shown that dormancy maintenance occurs because blocked cell-cell communication through plasmodesmata disallows growth-promoting signal transport into the SAM, and only after prolonged exposure to low temperature do plasmodesmata reopen, allowing access of the SAM to growth-promoting signals, after which active regrowth resumes (Tylewicz et al., 2018). *PpGH17* genes may respond to low temperatures because that low-temperature responsive elements were identified in 22 *PpGH17* genes. In addition, most *PpGH17* genes were induced during the transition from endodormancy to ecocormancy in apricot and peach due to chilling accumulation (**Figure 6**), which is consistent with the dormancy process in which adequate chilling induces dormancy release. A study in sweet cherry found that mainly of differentially expressed *GH17* genes repressed during endodormancy and highly expressed after dormancy release (Fouché et al., 2023), which was consistent with our study. The upregulation of *GH17* genes during dormancy release is critical for degrading callose in the neck regions of plasmodesmata and restoring intercellular communication in this stage, and after dormancy release and entry into the ecodormancy stage, excessive β-1,3-glucanase may not be needed; thus, these *PpGH17* genes are downregulated. However, some *PpGH17* genes are downregulated during chilling accumulation, and similar results have also been reported in poplar, in which some *GH17* genes are upregulated by chilling, whereas others are downregulated by chilling, suggesting that these proteins are not part of a chilling-induced dormancy release mechanism or the callose deposition and degradation remained necessary during this period (Rinne et al., 2011; Fouché et al., 2023).

In addition, studies have demonstrated that GH17 can be transported to the cell wall to play a role during dormancy release, especially for γ-clade members, which may be associated with lipid bodies. Chilling may recruit GH17 members locally in the dormant bud to hydrolyze plasmodesmata callose, in which GH17 proteins can be carried by lipid bodies displaced to the cell walls in close proximity to callose deposits at plasmodesmata during chilling, thus restoring cell-to-cell symplastic trafficking (Rinne et al., 2001, 2011; Veerabagu et al., 2020; Sheng et al., 2023). A study in grape revealed that the cell wall of buds significantly changed after hydrogen cyanamide (HC) treatment, one of the available exogenous dormancy releasers that shows promise for replacing chilling to break bud dormancy, accompanied by the upregulated expression of some VvGLUs and increased β-1,3-glucanase activity. Among these genes, a γ-clade β-1,3-glucanase, VvGLU1, was initially localized in the endoplasmic reticulum, accumulated in the vacuole, and was secreted into the cell wall during HC-triggered dormancy release (Shi et al., 2025). Thus, dormancy release requires not only the transcription of *PpGH17* genes but also the transport of the encoded proteins to their intracellular destinations. The cooperation of *PpGH17* genes with different expression trends may also maintain the balance of β-1,3-glucanase activity and regulate plasmodesmata permeability in the SAM of dormant buds. In addition, the composition of plasmodesmata is complex and regulated by many proteins, and β-1,3-glucanase is only one of the proteins associated with plasmodesmatal trafficking (Zanini et al., 2024). Therefore, *PpGH17* genes regulate plasmodesmata reopening together with other genes associated with plasmodesmata permeability in the dormancy release stage.

After dormancy release, the reopened plasmodesmata enable the movement of growth-promoting signals and drive bud burst when the temperature increases. A study in birch (*Betula pubescens*) suggested that the recovery of symplastic connectivity in spring is likely mediated by 1,3-β-glucanases (Rinne et al., 2001). The expression patterns of the *PpGH17* genes during ecodormancy-regrowth in our study indicating that *PpGH17* genes play roles in regulating bud regrowth, and the *cis*-elements linked to meristem activity and cell cycle regulation suggest PpGH17s may promote cell proliferation and tissue regrowth, which are important for dormancy release and bud-break. But there are few reports on whether GH17 is involved in the regulation of the bud-break process. In walnut buds, the activities of cell wall hydrolytic enzymes, such as pectin and hemicellulose, increase during bud break (Gholizadeh et al., 2021). The molecular regulatory mechanism of bud break is still unclear, and EBB1 is considered to be a conserved positive regulator of bud break in perennial woody deciduous plants (Yordanov et al., 2014; Zhao et al., 2020). Our previous study revealed that an earlier bud break in *PpEBB1*-overexpressing peach is linked to altered expression of many genes associated with cell wall modification (Zhao et al., 2020). Among the eleven *PpGH17* genes contained a GCC box-like element, four presented altered expression during the bud-break stage, indicating that they may participate in bud-break regulation via an EBB1-regulatory pathway. The interaction between PpKINβ2 and PpGH17–8 suggested that *PpGH17–8* may be regulated by phosphorylation. As for PpMIEL1, besides interacting with PpGH17-8, it also interacts with PpEBB1 as reported in our previous study (Zhao et al., 2021), in addition, the expression level of *PpGH17–8* was increased during bud-break stage, which was consistent with PpEBB1, indicating that PpMIEL1 may link the function of PpEBB1 and PpGH17–8 in regulating bud-break.

Bud dormancy is an important adaptive and economic trait for the survival and fruit production of deciduous fruit trees. An increasing number of studies begin to focus on the regulation of bud dormancy by cell wall modification, however, the function of *GH17* genes in bud dormancy regulation especially in peach is still not clear. Our study provides new insights into bud dormancy regulation in peach, and will help to identify potential key genes for dormancy-related molecular breeding and genetic engineering.

## Data Availability

The original contributions presented in the study are included in the article/**Supplementary Material**. Further inquiries can be directed to the corresponding authors.
