# Supplementary material for: Genome-wide identification of GH17 family genes and their expression patterns associated with bud dormancy-regrowth regulation in Prunus persica
Source: Front Plant Sci. 2025 Oct 8;16:1693135. doi: 10.3389/fpls.2025.1693135 (PMC12540407; doi:10.3389/fpls.2025.1693135)
Supplement: Supplementary file 1 [file DataSheet1.zip › Supplementary Files/Supplementary Figure 1.docx]

Supplementary Figure 1. Relative expression of *PpGH17*genes in peach buds from dormancy to the bud-break stage.
